# Subcellular localisation and identification of single atoms using quantitative scanning transmission electron microscopy

**DOI:** 10.1111/jmi.13410

**Published:** 2025-04-15

**Authors:** A. A. Sheader, G Vizcay‐Barrena, R. A. Fleck, S. J. L. Flatters, P. D. Nellist

**Affiliations:** ^1^ Department of Physics University of Oxford Oxford UK; ^2^ Centre for Ultrastructural Imaging King's College London London UK; ^3^ Wolfson Centre for Age‐Related Diseases Institute of Psychiatry Psychology and Neuroscience King's College London London UK; ^4^ Department of Materials University of Oxford Oxford UK

**Keywords:** dorsal root ganglia, high‐angle annular dark field imaging, neuron, oxaliplatin, scanning transmission electron microscopy, single atom imaging

## Abstract

Determining the concentration of elements in subcellular structures poses a significant challenge. By locating an elemental species at high spatial resolution and with subcellular context, and subsequently quantifying it on an absolute scale, new information about cellular function can be revealed. Such measurements have not as yet been realised with existing techniques due to limitations on spatial resolution and inherent difficulties in detecting elements present in low concentrations. In this paper, we use scanning transmission electron microscopy (STEM) to establish a methodology for localising and quantifying high‐*Z* elements in a biological setting by measuring elastic electron scattering. We demonstrate platinum (Pt) deposition within neuronal cell bodies following in vivo administration of the Pt‐based chemotherapeutic oxaliplatin to validate this novel methodology. For the first time, individual Pt atoms and nanoscale Pt clusters are shown within subcellular structures. Quantitative measurements of elastic electron scattering are used to determine absolute numbers of Pt atoms in each cluster. Cluster density is calculated on an atoms‐per‐cubic‐nanometre scale, and used to show clusters form with densities below that of metallic Pt. By considering STEM partial scattering cross‐sections, we determine that this new approach to subcellular elemental detection may be applicable to elements as light as sodium.

**LAY DESCRIPTION**: Heterogeneous elemental distributions drive fundamental biological processes within cells. While carbon, hydrogen, oxygen and nitrogen comprise by far the majority of living matter, concentrations and locations of more than a dozen other species must also be tightly controlled to ensure normal cell function. Oxaliplatin is a first‐line and adjuvant treatment for colorectal cancer. However, pain in the body's extremities (fingers and toes) significantly impairs clinical usage as this serious and persistent side effect impacts on both patient cancer care and quality of life. Annular dark‐field (ADF) imaging in the scanning transmission electron microscope (STEM) provides an image with strong atom‐number contrast and is sufficient to distinguish between different cell types and different organelles within the cells of the DRG. We also show that Pt may be imaged at the single atom level and be localised at very high resolution while still preserving a degree of ultrastructural context. The intrinsic image contrast generated is sufficient to identify these features without the need for heavy metal stains and other extensive processing steps which risk disturbing native platinum distributions within the tissue. We subsequently demonstrate that by considering the total elastic scattering intensity generated by nanometre‐sized Pt aggregations within the cell, the ADF STEM may be used to make a measurement of local concentration of Pt in units of atoms per cubic nanometre. We further estimate the minimum atomic number required to visualise single atoms in this setting, concluding that in similar samples it may be possible to detect species as light as sodium with atomic sensitivity.

## 1 INTRODUCTION

Heterogeneous elemental distributions drive fundamental biological processes within cells. While carbon, hydrogen, oxygen and nitrogen comprise by far the majority of living matter, concentrations and locations of more than a dozen other species must also be tightly controlled to ensure normal cell function.^1^ As abrupt variations in the local chemical landscape drive fundamental cellular processes, experimental methods which provide information about such elemental distributions are critical for understanding cell biochemistry. Those methods based on direct imaging, where chemical information may be acquired with ultrastructural context are of particular importance.^2^ Such techniques may be broadly categorised in terms of which physical mechanism is employed in the chemical analysis. One route is via examination of mass‐to‐charge ratios, as in laser‐assisted inductively‐coupled plasma mass spectrometry (LA‐ICP‐MS) and nanoscale secondary‐ion mass spectrometry (nanoSIMS).^3^ Alternatively, irradiation of a sample with high‐energy particles generates x‐rays with energies characteristic of the atomic species present. Analysis of these x‐rays is the foundation of techniques such as particle‐induced x‐ray emission (PIXE),^4^ energy dispersive x‐ray spectroscopy (EDX)^5^ in the electron microscope, and synchotron x‐ray fluorescence (SXRF).^6^

Fundamental detection limits and elemental specificity vary between chemical imaging methods.^7^, ^8^ Similarly, limitations on spatial resolution also differ. However, while some techniques are able to offer extremely high sensitivity and/or specificity, few options exist for the acquisition of data at the nanoscale within the context of cell ultrastructure. Only EDX is capable of recording chemical maps with resolution better than a few tens of nanometres; however, limitations on the electron dose tolerated by biological specimens mean the spatial resolution achievable in practice is often much poorer. Analysis of EDX data is also complicated in the complex multielement environment of the cell. In this case, overlapping characteristic x‐ray peaks can make quantification unreliable or even impossible when signal‐to‐noise is limited at high spatial resolutions. In this case, overlapping characteristic x‐ray peaks can make quantification unreliable or even impossible. Furthermore, at very high resolution the subtle effects of element extraction or redistribution during sample preparation become yet more problematic.^9^ Collectively, these factors mean that localising and quantifying low concentrations of elements at high resolution and with biological context is extremely challenging.

A number of recent developments in scanning transmission electron microscopy (STEM) imaging have shown promise for the high‐resolution chemical analysis of inorganic samples. In STEM, a focused beam of electrons is scanned across the sample. A post‐specimen annular dark‐field (ADF) detector is used to record the intensity of transmitted electrons scattered in a certain angular range. This intensity is subsequently plotted as a function of probe position to form an image. Unlike in the transmission electron microscope (TEM), a STEM‐ADF image recorded at sufficiently high angle is free from phase‐related effects.^10^ Image contrast is instead directly related to sample mass‐thickness, and therefore contains information about atomic number Z. This dependency means that it is possible to locate single atoms of heavy elements within low‐*Z* matrices.^11^, ^12^, ^13^ Progress in calibration of ADF detector sensitivity and collection angles^14^, ^15^ mean that it is now possible to unambiguously discern elemental species of single atoms from images alone.^16^, ^17^, ^18^

In this article, we show that quantitative ADF STEM methods may be adapted for subcellular elemental characterisation and used to localise single atoms of platinum (Pt) within neuronal cell bodies following in vivo administration of the Pt‐based chemotherapeutic oxaliplatin. Oxaliplatin is a first‐line and adjuvant treatment for colorectal cancer.^19^ However, chemotherapy‐induced peripheral neurotoxicity (CIPN) significantly impairs clinical usage as this serious and persistent side effect impacts on both patient cancer care and quality of life.^20^ While mass spectrometry studies on homogenised whole tissues have shown that platinum accumulates primarily in the dorsal root ganglia (DRG),^21^, ^22^ no current mass‐spectrometry instrumentation is available to identify the specific subcellular sites of drug deposition. Unknown sites of subcellular drug deposition limit understanding of the causal mechanisms of CIPN and thus hampers development of treatments to counteract neurotoxicity. The establishment of methods to characterise elemental distributions using in vivo models are therefore important for understanding Pt localisation in the DRG following systemic oxaliplatin treatment, and more broadly in cases where nanoscale elemental distributions drive interesting biological processes.

Within the STEM, ADF image contrast is sufficient to distinguish between different cell types and different organelles within the cells of the DRG. We show that Pt may be localised at very high resolution while still preserving a degree of ultrastructural context. The intrinsic image contrast generated is sufficient to identify these features without the need for heavy metal stains and other extensive processing steps which risk disturbing native platinum distributions within the tissue.^23^ We subsequently demonstrate that by considering the total elastic scattering intensity generated by nanometre‐sized Pt aggregations within the cell, ADF STEM may be used to make a measurement of local concentration of Pt in units of atoms per cubic nanometre. We further estimate the minimum atomic number required to visualise single atoms in this setting, concluding that in similar samples it may be possible to detect species as light as sodium with atomic sensitivity. The approach described here therefore has the potential to detect elements which are both present at low concentration and highly localised in particular subcellular compartments. This ability to combine high‐resolution imaging with the acquisition of chemical information unlocks the capability to investigate in vivo the chemical environment and determine drug deposition within cells.

## 2 METHODS

### 2.1 Animals

Adult male Sprague‐Dawley rats (starting weight 180–220 g; Harlan, UK) were kept in a climate‐controlled room containing rats only. Rats were housed in plastic cages with sawdust bedding and environmental enrichment materials. Bedding/cages were changed twice a week. Artificial light was provided on a 12 h light‐dark cycle (lights on at 7 am) with standard food/water ad libitum. All studies were carried out in accordance with the UK Animals (Scientific Procedures) Act, 1986. Protocols were approved by the Ethical Review Panel of King's College London and conducted under the UK Home Office project license 70/8015.

### 2.2 Oxaliplatin administration

Oxaliplatin was prepared from clinically formulated solution (Oxaliplatin Concentrate for Solution for Injection; 5 mg/mL; Accord Healthcare). Rats received systemic (intraperitoneal), intermittent administration of oxaliplatin over a 14‐day period, resulting in a cumulative dose of 25 mg/kg oxaliplatin. Rats were routinely visually checked and weighed to ensure good health.

### 2.3 Tissue extraction

Five hours after the last administration of oxaliplatin, rats received an overdose of pentobarbital. L3‐L5 DRG were then dissected bilaterally, with care taken to remove the ventral root. At room temperature, DRG were initially placed into phosphate‐buffered solution. This was followed by exposure to 0.5% formaldehyde fixative solution overnight at 4°C.

### 2.4 Cryosectioning

0.5% formaldehyde fixative solution provided sufficient protection to the cells and tissues to avoid ultrastructural changes caused by osmotic pressures during cryoprotection with high molarity sucrose solution. Higher concentrations and additional fixation using gluteraldehyde were avoided to lessen the likelihood of drug redistribution and extraction. Introduction into 2.3 M sucrose solution supports vitrification of the tissue upon plunge freezing directly into liquid nitrogen, preventing ultrastructural damage caused by ice crystal formation and cryodehydrative stress from extracellular ice formation.^9^

Ultrathin cryosections were cut using the Leica EM UC7 with FC7 cryochamber. Sectioning the DRG at –110°C with cutting thicknesses of 70–100 nm and cutting speeds of 3–5 mm/s produced acceptably thin electron transparent sections. Sections were transferred to pioloform‐coated copper TEM grids using a 50:50 1% methylcellulose‐2.3 M sucrose solution. The sections were then floated on distilled water droplets, before being washed for a total of 10 min per grid in fresh water droplets, changed every 2 min. The samples were then stored in low vacuum in a manually pumped storage container until examined in the STEM.

### 2.5 ADF STEM

**FIGURE 1:**
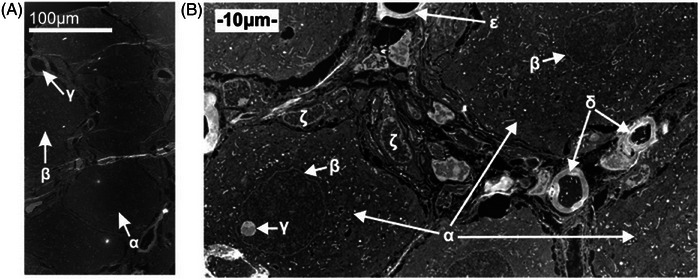
Low‐magnification ADF images recorded from a thin section of DRG allows for identification of regions of interest. The sample exhibits good ultrastructural preservation. (A) This image facilitate the detection of sensory neuronal cell bodies (α, β) and myelinated axons (γ). (B) A higher magnification allows observation of neuronal cell bodies (α), nuclear membranes (β), nucleoli (γ), small myelinated axons (δ), capillaries (ε) and other cells within the DRG (ζ).

**FIGURE 2:**
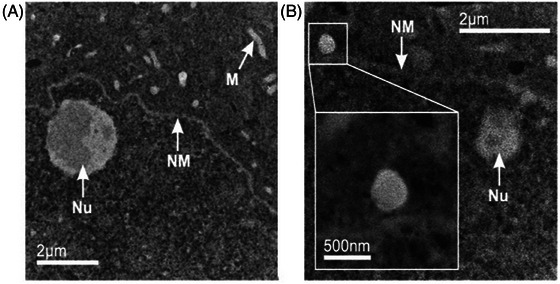
(A) Higher‐magnification ADF images show identifiable subcellular features such as mitochondria (M), the nucleolus (Nu) and nuclear membrane (NM). (B) Some regions in the DRG cytosol exhibit increased image contrast, correlating to higher average atomic number or density in that region. The inset shows an example of such a region on the exterior of the nuclear membrane which was subsequently found to contain Pt. The electron dose used to acquire images of the type displayed in (B) was on the order of 20–200 electrons per Å^2^.

Low‐magnification STEM survey images of the type shown in Figures 1 and 2 were obtained using a JEOL JEM ARM‐200F operated at 200 kV. As the microscope has a cold field emission electron source, and the probe‐forming optical system is corrected for spherical aberration, it is also appropriate for high‐resolution work. Instrument properties, such as probe convergence semi‐angle (α) and ADF detector collection angles were calibrated using the methods of Jones et al.^15^ The second largest condenser aperture was used to produce a probe semi‐angle of 22.4 mrad with a probe current of 80 pA. Typical dwell times and scan step sizes were 80 µs and 14 pm respectively . A camera length of 8 cm results in the ADF detector used in this experiment having an inner collection semi‐angle of 72.4 mrad, and an outer angle of approximately 271.0 mrad. These conditions are compatible with the ‘high‐angle’ regimen whereby signal collected for imaging is free from phase effects. Accordingly, the ADF image intensity generated varies as approximately Z^1.7^.^10^ For a biological specimen, such as the sectioned DRG examined in this work, this results in organelles with a higher average density or higher average atomic number scattering incident electrons more strongly, and thus appearing brighter in the final image. Although care had to be taken around electron‐beam damage to the sample, other beam‐induced effects, such as charging, were not observed.

### 2.6 EDX

**FIGURE 3:**
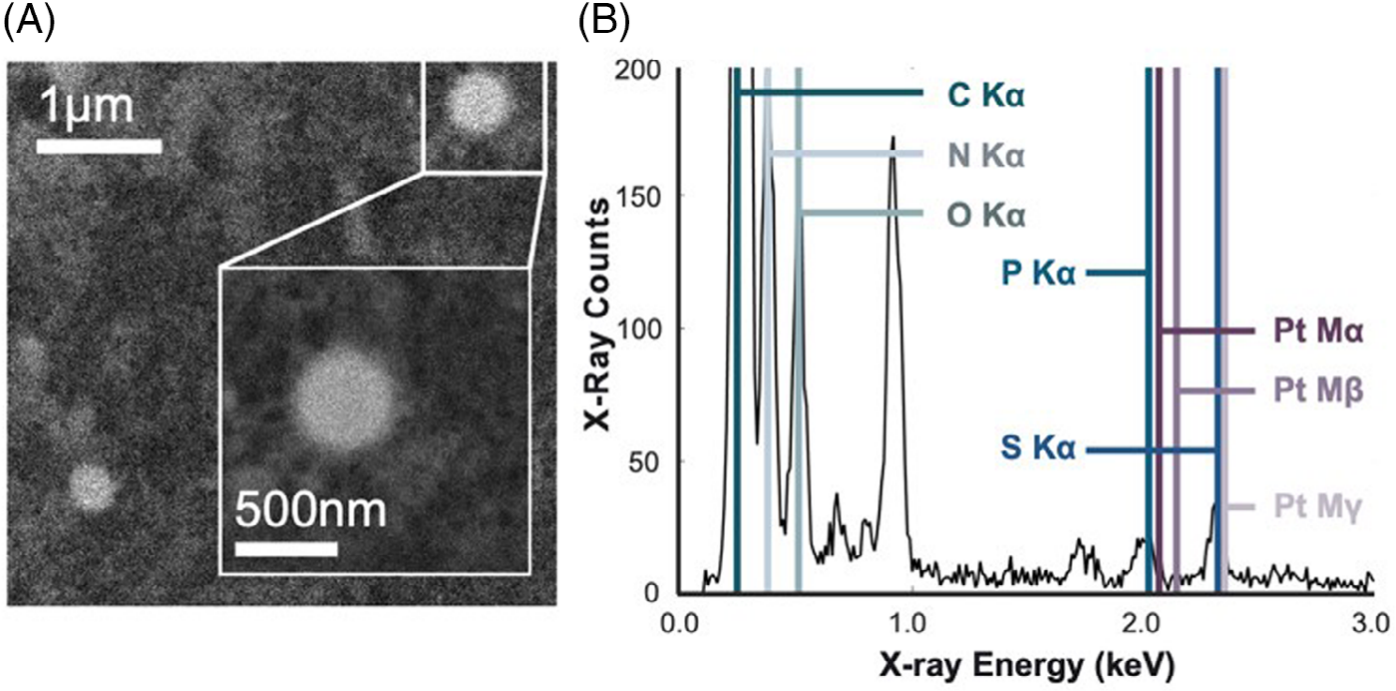
EDX spectrum acquired from Pt‐containing clusters in DRG. (A) Features similar to those in Figure 2 are visible throughout the DRG. (B) EDX acquired from continuously scanning over the inset region shows characteristic Pt x‐rays appearing as shoulders on the nearby P Kα and S Kα peaks. Due to limitations on electron dose, these peaks are at most only slightly above the spectral noise level. Qualitatively, the spectrum shows the sample is mostly comprised of carbon, nitrogen, oxygen, with trace amounts of other elements.

The EDX data shown in Figure 3 were obtained using a 100 mm^2^ JEOL detector. Typically in the STEM, it is possible perform spectral imaging (SI), whereby x‐ray data is collected point‐by‐point (in the same manner as the ADF image), resulting in an elemental map of that area. We found that the sample was unable to tolerate even the fastest SI acquisition conditions available on our instrument (0.001 s/pixel). In comparison, the fastest imaging condition is 1.7μs – that is, nearly 600 times faster than the SI. We found that by continuously recording EDX data while keeping the electron probe moving (by continually scanning), it was possible to record some EDX spectra with limited counts over a period of about 500 s. Even when probe current was significantly reduced, the sample was still unable to tolerate long periods of continual irradiation. Detection of nanoscale clusters within a biological specimen under such conditions would therefore be impossible, as it would not be possible to collect sufficient signal before significant damage to the specimen.

### 2.7 Depth of focus

The depth of focus in a STEM image is given by

$$\Delta z=1.77\frac{\lambda }{\alpha^{2}},$$

for a given incident electron wavelength λ (approximately 2.5pm at 200 kV).^24^ The value of Δz is therefore approximately 9 nm. Single atoms were identified by repeatedly sweeping through the thickness of the section. In some cases, this resulted in a significant amount of beam‐induced motion for particular atoms. While motion in the beam direction does not affect the measured *σ_ADF_*, motion in the scan direction is problematic. Atoms which were visibly distorted and appeared non‐symmetric and non‐pointlike were therefore excluded from the final analysis.

### 2.8 Image simulation

Multislice STEM ADF image simulations of single atoms with different atomic numbers were performed with the MULTEM v2.2.1 code, using the frozen phonon model and the Lobato potential parameterisation optimised for high‐angle simulations.^25^ The microscope accelerating voltage, probe convergence angle and ADF collection angles used in the simulations were matched to experimental values. As ADF STEM cross‐sections are robust to aberrations,^26^ effects of defocus and higher order aberrations were ignored. Single atoms were placed at the centre of the 30 × 30 nm simulation grid, which was sampled at 0.029 nm intervals. This sampling was sufficient to capture the full high‐angle scattering within the simulation.

### 2.9 Pt cluster weighing

Cluster weighing was achieved by comparing the experimental value of a Pt single‐atom cross section with the total cluster cross section. For a cluster of *N_atoms_*, the cluster mass *m* may be obtained through the formula

$$m=M_{m}\;\frac{N_{atoms}}{N_{A}},$$

where *N_A_* is Avagadro's number (6.02 × 10^23^ mol^−1^) and *M_m_* is the mass of one mole of a given element. For platinum, *M_m_* is approximately 195 .08 g. Atoms per cubic nanometre values are calculated assuming a spherical cluster volume. The largest contributions to error in this measurement arise from beam‐induced drift during the imaging process, and the accuracy in accounting for a locally varying background. Challenges relating to the locally varying background, which may arise from out‐of‐focus Pt atoms, were addressed by subtracting a value calculated from only the most proximal pixels in a 10×10 pixel window around each individual atom. The average cluster size is 3.5 nm (*n* = 12, *s* = 0.7 nm).

## 3 RESULTS

### 3.1 Dorsal root ganglia preparation for STEM

High‐resolution transmission electron microscopy necessitates the preparation of an ultrathin electron‐transparent sample, capable of withstanding both high vacuum and high levels of electron irradiation. In conventional electron microscopy, samples are often heavily fixed to achieve sufficient stability in the microscope and/or stained using heavy metals to increase the image contrast.^23^ Unfortunately, such methods are broadly incompatible with elemental analysis due to the risk of extraction and redistribution.^9^, ^27^, ^28^ To avoid these potential complications, we developed a sample preparation approach based on the Tokuyasu immunolabelling method.^29^ At room temperature, DRG were initially placed into phosphate‐buffered solution. This was followed by exposure to 0.5% formaldehyde fixative solution overnight at 4°C. Following fixation, the DRG were then placed in a 2.3 M sucrose solution before being plunge frozen and stored in liquid nitrogen.

Whole rat DRG were recovered following prolonged in vivo oxaliplatin administration. Following light fixation overnight using a 0.5% formaldehyde solution, the DRG were placed in a 2.3 M sucrose solution before being plunge frozen and stored in liquid nitrogen. Ultrathin samples of frozen DRG were then sectioned by cryoultramicrotomy. Sections were transferred to pioloform‐coated copper TEM grids using a 50:50 1% methylcellulose‐2.3 M sucrose solution, which acted to embed the sample in a thin polymer layer. Sections and grids were subsequently thawed on the bench to produce high‐quality samples with identifiable ultrastructure which were stable at room temperature. Full details concerning oxaliplatin administration, dissection, freezing and cryosectioning may be found in the Supporting Information.

### 3.2 Identification of regions of interest

Screening samples using low‐magnification ADF STEM allowed rapid identification of regions containing DRG neuronal cell bodies (Figure 1). The image contrast generated by the unstained sample was sufficient to distinguish different organelles such as nuclei and mitochondria, along with other structures such as the nuclear membrane and nucleolus (Figure 1B). In some cell bodies other features (similar to those indicated in Figure 2) were not immediately identifiable as specific organelles. However, the increase in image intensity at these sites was indicative of more high‐angle scattering arriving at the ADF detector, and therefore associated with a higher average density and/or atomic number and thus Pt accumulation.

Attempts to use EDX to confirm the presence of Pt in such features were unsuccessful due to insufficient x‐ray count rates. The example EDX spectrum in Figure 3 was produced by summing EDX signal while continually scanning over the Pt‐containing feature observed in the cell cytoplasm. Qualitatively, the spectrum shows the feature is mostly comprised carbon, nitrogen, oxygen. Smaller peaks correspond to detection of elements present in much smaller amounts.

For Pt, the strongest corresponding x‐ray peak is expected at 2.05 keV. However, this peak is convolved with the phosphor x‐ray peak at 2.01 keV. As the spectrometer resolution is only around 140 eV, any Pt signal appears at most as a small shoulder on the primary peak. It is not possible to resolve the Pt signal further as EDX counts were severely limited by the electron dose‐tolerance of the sample.

### 3.3 Atomic resolution imaging

**FIGURE 4:**
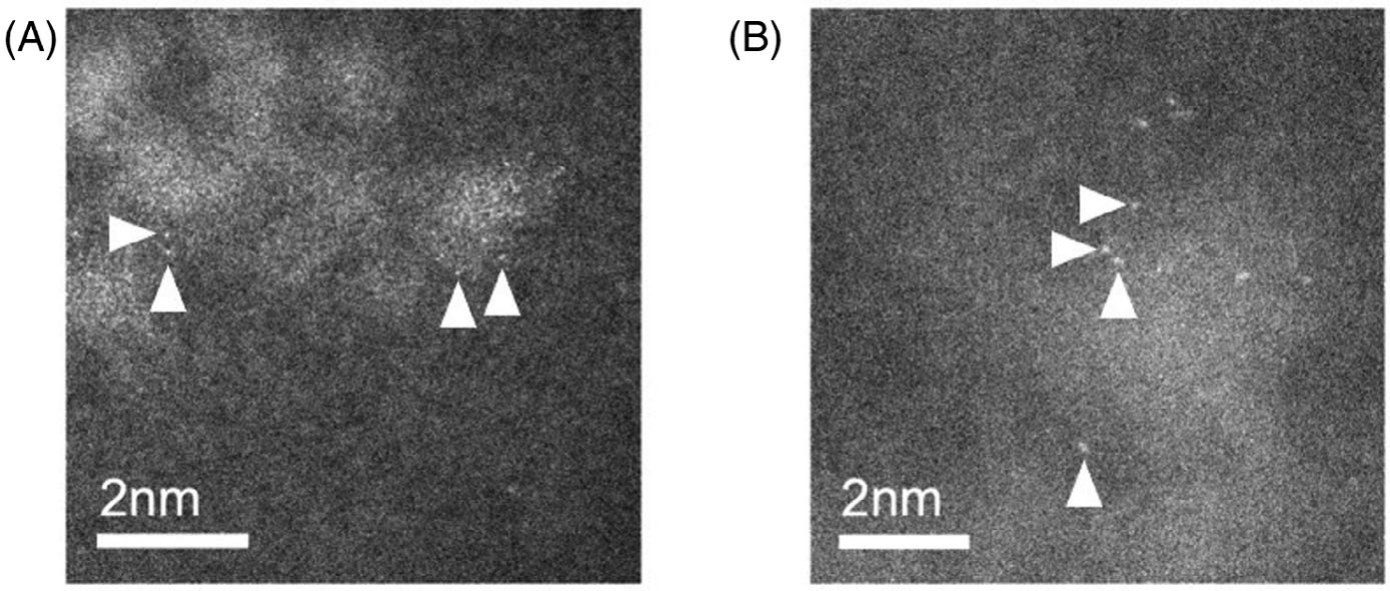
ADF images of atomic clusters located using high‐magnification STEM. Single atoms are identified by arrowheads.

Further imaging within high‐intensity features in some cases showed internal structure. As shown in Figure 4, at sufficiently high magnification, it was possible to observe small clusters of approximately a few nanometres in diameter and similar to those observed previously in oxaliplatin.^30^

**FIGURE 5:**
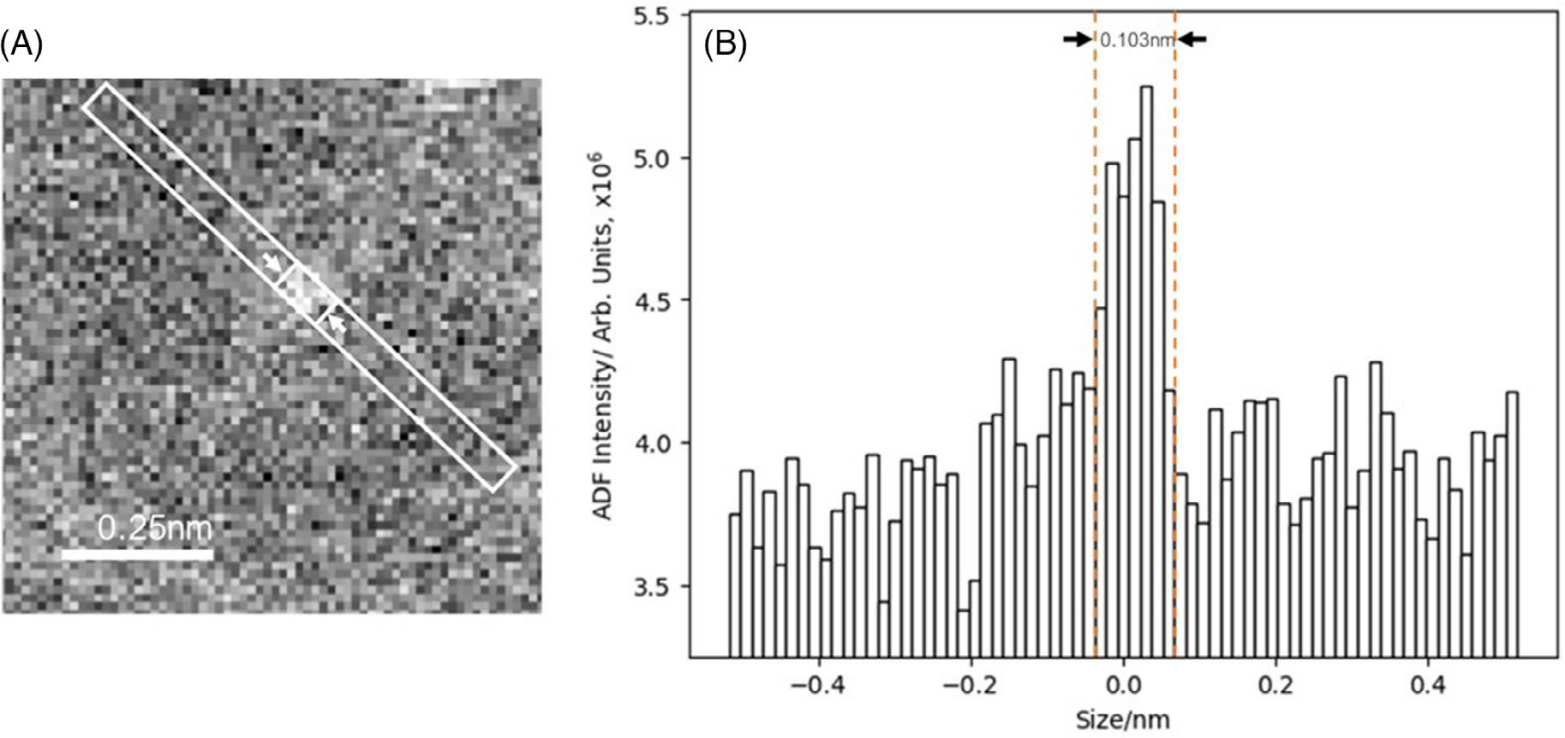
Image intensity line profile demonstrating the ångström (Å)‐scale of the single‐atom peaks.

It was further possible to resolve single atoms within these structures, identifiable in Figure 4 as ∼1 ångström (Å)‐scale intensity peaks in the images (Figure 5). The remainder of the cluster is out of focus due to the relatively large numerical aperture required to image single atoms. Under these conditions, depth of focus is restricted to around 9 nm. As this is significantly smaller than the thickness of the sample, the focal point of the electron probe was repeatedly swept through the full depth of the section to locate single atoms.

The total electron fluence required to achieve single atom imaging was approximately 1×10^6^ electrons/Å^2^. This value is several orders of magnitude higher than typically used for biological imaging, but is necessary to generate sufficient signal to distinguish single atoms above the background noise level and reliably identify their species from ADF images. However, its use was restricted to the imaging of single atoms and atomic clusters only and therefore did not compromise the ultrastructure observable at lower magnifications. Images formed over a large scan areas (such as those shown in Figure 1) expose the sample to very little irradiation and are therefore unlikely to perturb cell ultrastructure.

### 3.4 Species identification from elastic scattering strength

**FIGURE 6:**
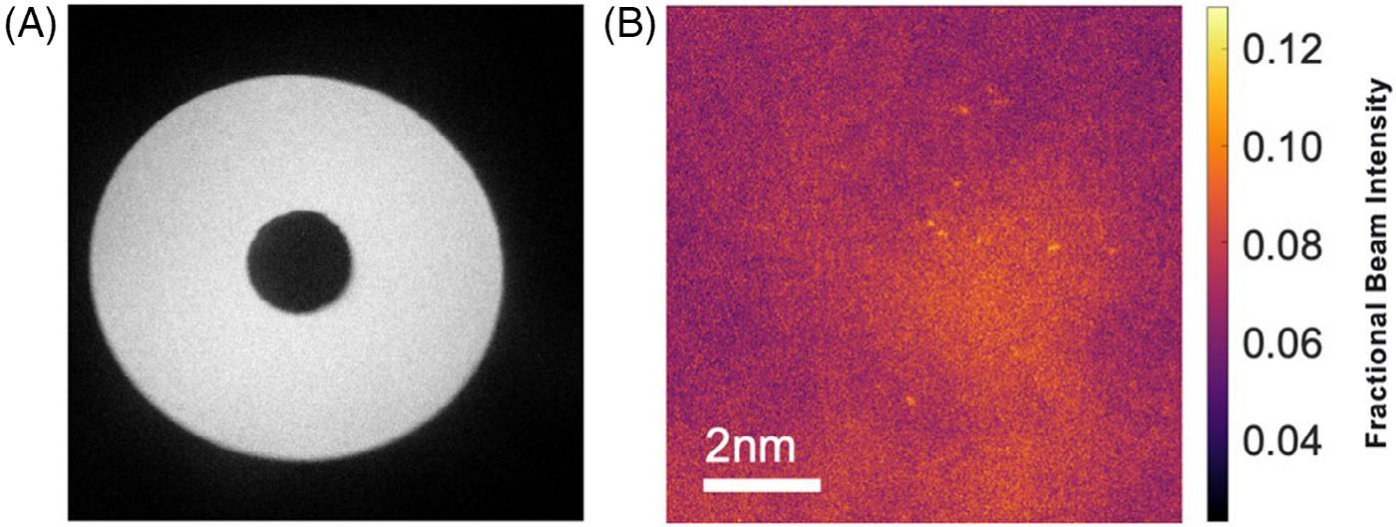
(A) Response map of the upper ADF detector in the Oxford JEOL ARM‐200F. Non‐uniform intensity indicates the response is not homogeneous across the detector. (B) Atomic cluster image converted to units of fractional beam intensity. The most intense areas indicate as much as 12% of the beam is scattered to the ADF detector at some probe positions.

As the fraction of the incident electron beam scattered by an atom is characteristic of its atomic number, by carefully measuring this value it is possible to identify its elemental species from images of the type shown in Figure 4. The success of the quantification procedure used in this paper relies upon accurate measurements of the ADF detector parameters. First, the detector response and inner and outer angular radius were calibrated following the procedure outlined by Jones et al.^15^ The value of a given pixel in the image $I_{frac}$ for position vector R, then represents the fraction of the incident electron beam that is scattered to the detector for the corresponding illuminating probe position through the equation

(1) $$I_{frac}\left(R\right)=\frac{I_{exp}\left(R\right)-I_{vac}}{I_{det}-I_{vac}}\;$$

where $I_{exp}$ is the intensity in the pixel from the experiment, $I_{vac}$ is the background electronic noise intensity level recorded in vacuum, and $I_{det}$ is a radially averaged value of detector response when illuminated by the full unscattered beam. The values of $I_{vac}$ and $I_{det}$ were obtained by recording an ADF detector response sensitivity ‘map’ in a confocal manner; this was achieved by scanning the electron probe across the full ADF detector and recording the detector response at each scan position (Figure 6). This is necessary as the detector response is not uniform, and thus greater and lesser degrees of sensitivity at different points on the detector must be accounted for in order to very accurately quantify ADF images.^31^, ^32^ The experimental image and detector map were both analysed using the AbsoluteIntegrator v1.6.4 code.^33^ The results are shown in Figure 6, indicating that for a single platinum atom only a few per cent of the incident beam electrons are scattered to the ADF detector.

Placing single‐atom images on this absolute scale allows for direct comparison between experimental measurements of elastic scattering strength and those predicted by simulation. In particular, it is often desirable to measure a partial scattering cross‐section $\sigma_{ADF}$ for all or part of the image; this parameter has been shown to be robust to microscope aberrations, and suitable for identifying atomic species.^26^

Values of $\sigma_{ADF}$ may found through

(2) $$\;\sigma_{ADF}=\sum_{i}\;AI_{frac}^{\left(i\right)},$$

where A is the area of the STEM image pixel, and $I_{frac}$ is the fraction of the incident intensity scattered to the detector in pixel i. To identify the atomic species of a single atom, following an appropriate subtraction step to remove the contributions of any out‐of‐focus background, the summation is then made over all pixels associated with that atom. Care must be taken to ensure only atoms which remained stationary during imaging are examined; electron‐beam induced motion results in blurry or streaked atoms which cannot be characterised.

Cross sections are commonly expressed in units of barns, with 1 barn (b) equal to 10^−28^ m^2^. The final mean cross section measurement for the single atoms observed within the ∼3 nm clusters was $\sigma_{ADF}^{atom}=$1.15 Mb (for *n* = 13 single atoms, and standard deviation *s* = 0.03 Mb). ADF image simulations for single platinum atoms were performed with MULTEM^25^ and using inner and outer ADF collection angles measured experimentally. These simulations predicted a $\sigma_{ADF}^{atom}$ value for an isolated Pt atom of 1.13 Mb, in agreement with our result.

**FIGURE 7:**
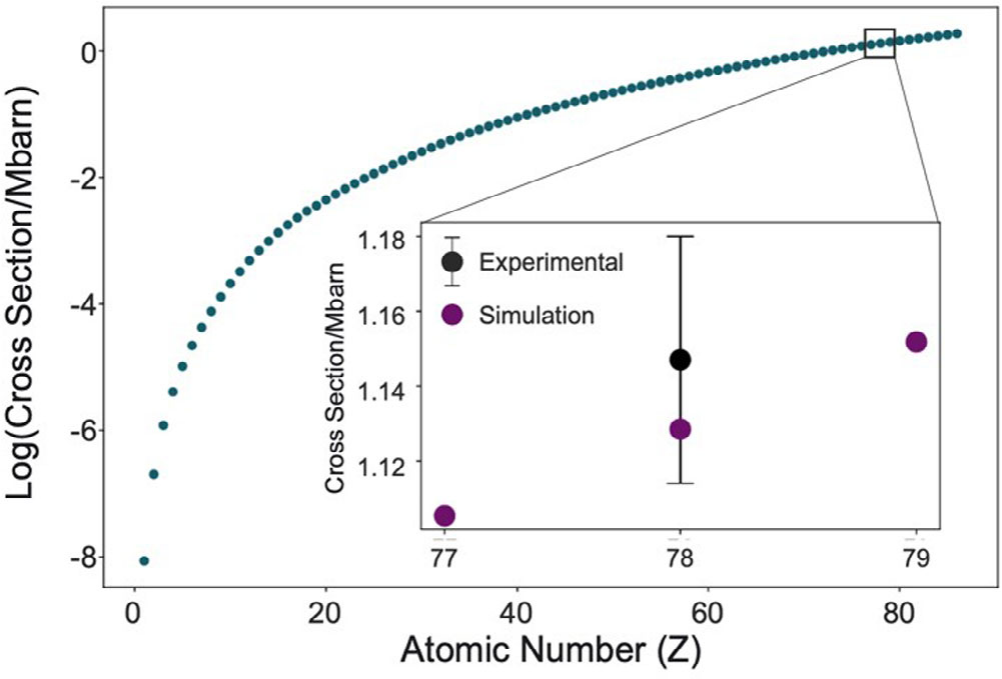
Logarithmic plot of simulated ADF scattering cross‐section against atomic number. The inset graph shows expected cross‐sections for iridium, platinum and gold (*Z* = 77, 78 and 79, respectively). Experimental data with an error bar of one standard deviation are also shown in black.

While this confirms that the single atoms we observed within the small clusters are platinum, for the avoidance of doubt, $\sigma_{ADF}^{atom}$ values were also calculated for other species. Figure 7 shows that predicted partial scattering cross section values for lighter and more biologically abundant elements are more than an order of magnitude smaller than measured from experimental images. The amount of high‐angle scattering generated by these single atoms is therefore consistent with a high‐Z element such as platinum, presumed to be present in the cell due to oxaliplatin administration.

### 3.5 Cluster weighing: highly localised mass measurement

Following successful identification of single platinum atoms within the DRG, it was then possible to use a similar approach to count the total number of Pt atoms in the small clusters observed at high magnification. Following a similar approach to the procedure for obtaining the single atom cross‐section, local background subtraction and image analysis in accordance with Equations (1) and (2) produced a ‘cross‐section per cluster’ value, $\sigma_{ADF}^{cluster}$. As ADF STEM is an incoherent imaging method, this measurement is linearly dependent on the total number of atoms $N_{atoms}$ in the cluster, in accordance with

(3) $$\;\sigma_{ADF}^{cluster}=N_{atoms}\;\times \sigma_{ADF}^{atom}$$

and could therefore be used to estimate local Pt concentration.

**FIGURE 8:**
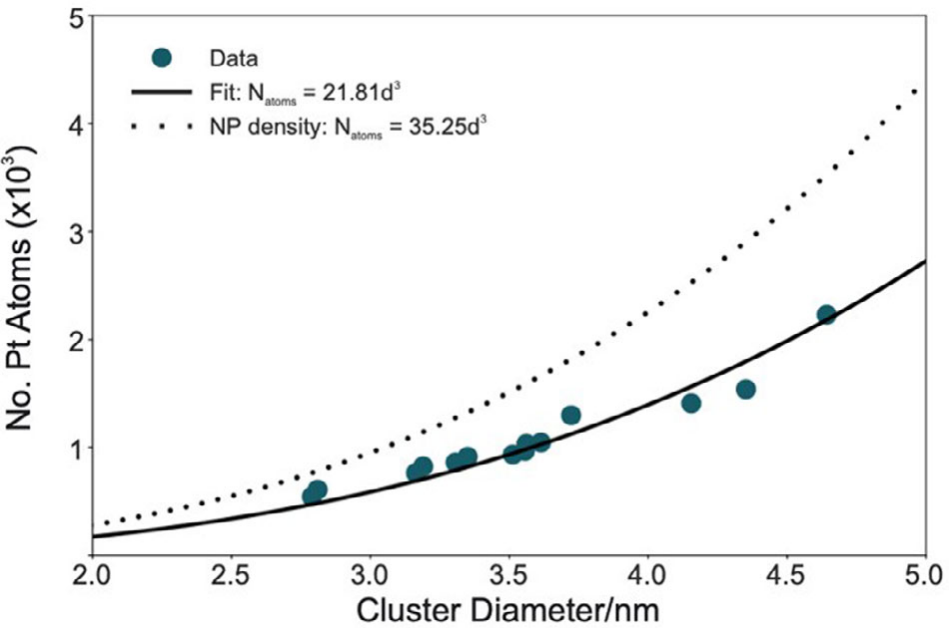
Atomic cluster weighing in the DRG. Experimental data points are fitted with a cubic dependence upon cluster diameter (solid line). The results of this fit indicate that Pt clusters found within the DRG contain approximately 40% fewer atoms per cubic nanometre when compared to an equivalently sized metallic Pt nanoparticle (dotted line). Within the DRG, the average cluster diameter is around 3.5 nm, and contains approximately 1000 atoms.

Figure 8 shows the estimated Pt mass from discrete clusters as a function of cluster diameter. By assuming clusters are approximately spherical, it could reasonably be expected that $N_{atoms}$ has a cubic dependency on diameter. By fitting experimental data using this dependency, we estimate Pt clusters found within the DRG have a density of around 60% that of metallic platinum nanoparticles, with the average cluster of diameter 3.5 nm containing around 1×10^3^ platinum atoms. This is equivalent to approximately 330 zeptograms of Pt, and a density of 5.2 atoms per cubic nanometre. The ability to discern density information in this way is therefore a particular strength of this technique, having additional capabilities to probe structural information about nanoscale aggregations.

### 3.6 Visibility of other species

Single atoms of platinum were readily visible in these tissue sections due to its high atomic number, and correspondingly large degree of high‐angle elastic scattering in the STEM. However in principle the methods described here may be applied more generally to the challenge of detecting and quantifying any elemental species, provided the signal generated by a single atom is sufficiently high. The number of electrons, $n_{el,x}$ scattered to the ADF detector by one atom of species x is given by

(4) $$\;n_{el,x}=D_{e}\;\sigma_{ADF,x}^{atom},$$

where $D_{e}$ is the electron fluence in units of electrons per area and $\sigma_{ADF,x}^{atom}$ is the single atom partial scattering cross section for that element. However, for a single atom embedded in a low‐Z matrix (such as in a thin tissue section) of element y the noise generated by the thickness of the sample also becomes important. The matrix signal is similarly given by

(5) $$\;n_{el,matrix}=D_{e}\;\sigma_{ADF,y}^{atom}A_{int}\rho_{y}t,$$

where $A_{int}$ is the integration area necessary to capture the full scattering of the single atom of interest, $\rho_{y}$ is the density of that element and t is the sample thickness. In the STEM, the noise $\Psi_{el}$ generated by both the single atom of interest and the matrix obeys Poissonian statistics^34^ and is given by

(6) $$\;\Psi_{el}=\sqrt{n_{el,matrix}+n_{el,x}}.$$

This noise is the limiting factor in determining species using the approach described here, and success is therefore a function of many parameters, including the electron dose tolerated by the sample, the atomic numbers of both the single atom and the matrix, the matrix density, sample thickness and detector collector angles. However, by making a number of sensible assumptions about the sample and experimental conditions, it is possible to estimate the minimum atomic number which might be quantifiable using ADF imaging.

**FIGURE 9:**
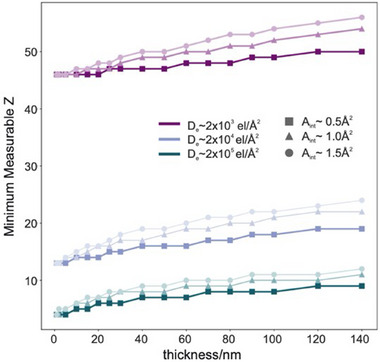
Minimum detectable atomic number of single atoms embedded in a low‐Z matrix. Detectability is strongly dependent upon electron fluence, but also upon sample thickness and image integration area.

Assuming a hydrated sample density of 1 g/cm^3^ and a 70% water content,^35^ the remaining 30% of the sample mass may be assumed to be pure carbon (in reality this likely overestimates the background contribution to the electron scattering). This means that each cubic nanometre of dehydrated sample contains approximately 15 carbon atoms. Figure 9 shows the estimated minimum quantifiable atomic number for single atoms as a function of sample (matrix) thicknesses using the simulated cross‐sections described for this experiment. We estimate that using an aberration‐corrected cold field emission gun STEM with an electron probe with full width at half maximum of about 80 pm, in a sample with nominal cryosectioning thickness of 70 nm we may expect to detect clustered single atoms of elements as light as sodium. In practice, it is likely that this this limit is degraded by other experimental factors. For example, elements which are present at naturally occurring low concentrations or those which are preferentially extracted during the sample preparation procedure may fall below levels which make it unlikely that single atoms are observed during the experiment. Alternatively, beam‐induced motion of light species results in $A_{int}$ increasing above the area of the electron probe. This would degrade the detectability limit and pose further challenges in discriminating atoms of similar *Z*. Nevertheless, this result is of potential benefit to the examination of lighter species in a biological setting. As nerve tissue contains relatively high concentrations of endogenous elements such as Na, K, Ca, Fe and Zn,^36^, ^37^, ^38^, ^39^ provided these were present at sufficiently high concentrations it may be possible to identify single atoms of these species in addition to Pt.

## 4 DISCUSSION AND CONCLUSIONS

We have developed a new methodology for locating heavy elements within cells at atomic resolution and with high fidelity. Measurements of elastic scattering strength allow both compositional and density information to be acquired for nanoscale elemental while retaining ultrastructural context. In common with other techniques used to examine elemental distributions in cells and tissue, use of the STEM requires the sample be processed sufficiently to generate a thin and vacuum‐robust specimen. However, despite avoiding strong fixation and staining, we have shown that the intrinsic ADF image contrast is sufficient to navigate ultrastructure and locate platinum within the cells of the DRG.

The quantification route used here is dependent on careful comparison of high‐resolution experimental and simulated data, which is only attainable using an aberration corrected STEM. However, the physical mechanisms which give rise to the ADF image contrast are universal for any STEM equipped with an annular detector. It would therefore be possible to measure the masses of small elemental clusters without knowledge of ADF detector geometry or image simulation, for example by using comparative measurements similar to those demonstrated for biological macromolecules.^40^, ^41^

This approach may be beneficial for quantitative high‐resolution studies where EDX spectra are impossible or impractical to acquire due to limitations on electron fluence. As shown here, characteristic x‐rays could not reliably distinguish Pt within the sample, and data were insufficient to quantify aggregates within the cell. This is a result of both limited x‐ray yield and the small size of the EDX detector. The inelastic electron‐sample interaction which generates x‐rays occurs with about two orders of magnitude lesser probability than the elastic scattering used to form an ADF image.^42^ Furthermore, microscope geometry typically restricts the EDX detector from collecting more than a few per cent of this signal. In comparison, the efficient collection of the more abundant elastically scattered signal by the ADF detector means that the partial scattering cross section for a given atom is up to 10^6^ times greater in ADF than EDX^43^ Recent improvements in EDX collection efficiencies are unlikely to overcome this difference. As electron microscopy on biological materials is often limited by the electron dose withstood by the sample, making use of a strongly generated signal is important to maximise the amount of information for the least amount of beam‐induced damage.^44^ Similar considerations are also important in cryo‐STEM, where single‐atom detection affords opportunities for understanding protein function and more broadly for protein labelling.^45^

While it is not immediately possible to draw direct conclusions about subcellular Pt binding and its role in CIPN from this work, the potential applications of high‐resolution electron microscopy to probe elemental aggregation over these length scales is readily apparent. Pt has been observed to accumulate in other tissues following treatment with different Pt‐based complexes. For example, nanoSIMS and LA‐ICP‐MS experiments have shown co‐localisation of Pt with sulphur‐rich organelles in germ cell tumours following the in vivo administration of the Pt‐based chemotheraputics cisplatin and carboplatin.^46^ Similarly, in vitro experiments have shown co‐localisation for Pt and S, with additional Pt accumulations in mitochondria, autophagosomes and the nucleoli of ovarian cancer cells.^47^ It is therefore likely that the platinum‐containing features reported here do not arise due to artefacts of the sample preparation procedure, and are instead related to the intracellular transport of oxaliplatin in neuronal cell bodies.

The limited spatial resolution of other techniques mean that thus far only electron microscopy (and in particular the high‐resolution ADF STEM methods described in this work) are capable of resolving details of Pt distributions at length scales better than a few tens of nanometres. Other Pt‐based chemotherapeutics have been shown to contain small metallic nanoparticles, both in their dried form^30^, ^48^ and in the presence of blood products.^49^ Examination of the fate of these nanoparticles in vivo, including their concentration and aggregation, is an important motivation for the development of very high‐resolution methods for localising elements at the nanoscale as detailed in this work. In addition to aiding mechanistic understandings of CIPN, the techniques described here may also be applicable to the fields of nanotoxicology and pharmacology. Metal and metal oxide nanoparticles are now widely used in manufacturing, and are therefore inevitably present in the environment.^50^ Unfortunately such particles have been shown to have deleterious effects to human health, for example such as the potential neurodegenerative role played by iron‐rich pollutant nanoparticulates in the brain^51^ and the toxic sequestration of elements such as arsenic following exposure to contaminated water.^52^ The use of STEM methods to ascertain information about nanoscale elemental concentrations within cells can therefore help unlock novel opportunities for subcellular investigation.
